# Efficacy of azithromycin 1.5% eye drops in childhood ocular rosacea with phlyctenular blepharokeratoconjunctivitis

**DOI:** 10.1186/1869-5760-3-38

**Published:** 2013-02-22

**Authors:** Serge Doan, Eric Gabison, Frédéric Chiambaretta, Melissa Touati, Isabelle Cochereau

**Affiliations:** 1 Department of Ophthalmology, Hôpital Bichat and Fondation A. de Rothschild, 75018, Paris, France; 2 Department of Ophthalmology, Hôpital G Montpied, 63000, Clermont-Ferrand, France

**Keywords:** Rosacea, Children, Keratoconjunctivitis, Blepharitis, Azithromycin

## Abstract

**Background:**

The purpose of this study is to report the efficacy of azithromycin 1.5% eye drops in children with ocular rosacea and phlyctenular blepharokeratoconjunctivitis. This retrospective study from January 2009 to March 2010 included 16 children treated with lid hygiene plus azithromycin 1.5% eye drops (Azyter®): 3-day treatments (1 drop twice a day) every 10 days, reduced based on efficacy to one treatment every 15 days and then to one treatment per month.

**Results:**

Nineteen eyes of six boys and ten girls, aged 4 to 16 years (mean, 9.3 ± 4.0) were included. The disease was previously resistant to lid hygiene (all the patients), oral erythromycin (one patient), and intermittent topical steroids (six patients). The median duration of each phase of azithromycin treatment (i.e., three, two, and one treatments per month) was 2 months. Ocular inflammation was controlled by azithromycin alone in 15 patients. In one uncontrolled case, cyclosporine 2% eye drops was added at month 5. Bulbar conjunctival hyperemia resolved completely within 1 month in all eyes, whereas conjunctival phlyctenules and corneal inflammation took longer to improve, with a complete resolution within 3 to 10 months. Blepharitis grade decreased from 2.31 ± 0.79 to 1.50 ± 0.73. Treatment was stopped after a median of 6 months (from 4 to 10 months) without recurrence of corneoconjunctival inflammation (median follow-up without treatment, 11 months). Six cases of ocular irritation were reported, two of which led to treatment withdrawal.

**Conclusion:**

Azithromycin 1.5% eye drops is an effective treatment for phlyctenular keratoconjunctivitis complicating childhood ocular rosacea.

## Background

Childhood ocular rosacea is a rare but often misdiagnosed disorder associated with chronic blepharitis and, in many cases, phlyctenular keratoconjunctivitis [1]. It is alternatively named blepharokeratoconjunctivitis [2,3] or staphylococcal phlyctenular keratoconjunctivitis. Lid involvement is characterized by chronic posterior and anterior blepharitis with frequent chalazia. Ocular inflammation is often unilateral with chronic conjunctival hyperemia; phlyctenules on the conjunctiva, the limbus, or the cornea; and inferior punctate keratopathy. Occasionally, corneal involvement is more severe with predominantly inferior (sub) epithelial infiltrates, stromal ulcers, neovascularization, and scars, leading to visual sequelae in 17% of cases [4]. Skin involvement is inconstant.

The primary mechanism of childhood ocular rosacea is chronic meibomitis. Staphylococcal infection in the meibomian glands is probably secondary and may induce conjunctival and corneal inflammation by a T lymphocyte immune response against bacterial parietal antigens or toxins. The phlyctenules and corneal infiltrates are the result of a type IV cell-mediated late hypersensitivity reaction according to the Gell and Coombs' classification [5].

The treatment of childhood ocular rosacea is based on its presumed physiopathogenic mechanisms and involves improving meibomian gland function, controlling bacterial proliferation with lid hygiene and antibiotics, and inhibiting T cell-mediated inflammation with topical steroids or cyclosporine. Daily lid hygiene is the cornerstone of ocular rosacea treatment, with oral antibiotics required in case of lid hygiene failure or in severe forms of the disease. Second-generation tetracyclines are used in children over 8 years old, and erythromycin, azithromycin, or metronidazole are used in younger patients [6-8]. Topical antibiotics are often used, but very few publications support their efficacy [3]. Topical steroids are very effective but should only be used in case of acute corneal inflammation due to the risk of iatrogenic complications. Cyclosporine 0.5% to 2% eye drops is an interesting alternative in children with steroid-dependent disease [9].

Azithromycin is a macrolide antibiotic characterized by a broad antibacterial spectrum, a long half-life related to its tissue and cell penetration [10], and anti-inflammatory properties [11]. Oral azithromycin is an effective treatment in ocular rosacea [12] and also in cutaneous involvement of rosacea (with efficacy comparable to doxycycline) [13].

In this retrospective study, we assessed the efficacy of topical 1.5% azithromycin eye drops in children with ocular rosacea and phlyctenular blepharokeratoconjunctivitis.

## Results

Sixteen children (19 eyes) were treated: six boys and ten girls with a mean age at initiation of azithromycin treatment of 9.3 ± 4.0 years (range, 4 to 16 years).

### Childhood ocular rosacea status before azithromycin treatment

Pretreatment status is detailed for individual patients in Table 1. The disease was asymmetric or unilateral in 13 children. Mean interval between onset of the disease and azithromycin treatment was 39 months (from 8 months to 12 years). Two children had ongoing recurrent chalazia, whereas recurrent episodes of red eye were reported in all cases. Bulbar conjunctival hyperemia and phlyctenules were present in all eyes, and corneal inflammation was noted in all eyes but one. Therapy before azithromycin (which had not controlled the inflammation) consisted of lid hygiene in all cases plus oral erythromycin in one case and intermittent topical corticosteroids in six cases. Topical steroids and oral erythromycin were stopped in all cases.

**Table 1:** Patient demographics and baseline characteristics

**Patient no.**	**Sex**	**Age**	**Unilateral**	**Evolution (months)**	**Treatment**
1	F	10	Yes	96	LH
2	M	12	No	30	LH
3	F	7	Yes	15	LH + steroids
4	F	10	Yes	8	LH + steroids
5	F	7	Yes	15	LH
6	F	8	No	69	LH
7	M	15	Yes	18	LH + steroids
8	F	14	Yes	20	LH
9	F	6	Yes	10	LH + ERY
10	F	16	No	90	LH
11	M	5	Yes	12	LH + steroids
12	F	10	Yes	15	LH
13	M	5	Yes	27	LH + steroids
14	F	7	Yes	24	LH + steroids
15	M	4	Yes	20	LH
16	M	15	Yes	148	LH

LH, lid hygiene; ERY, oral erythromycin.

### Efficacy of azithromycin

**Figure 1:**
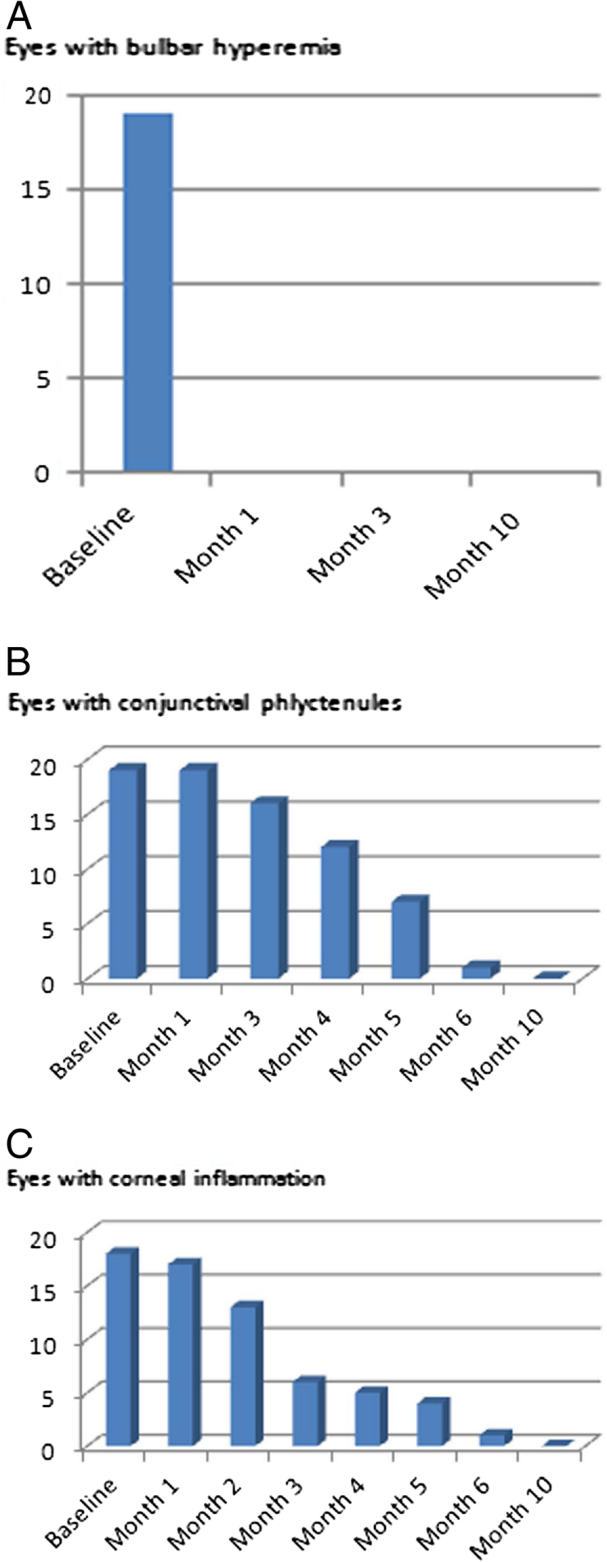
**Improvement of bulbar conjunctival hyperemia, conjunctival phlyctenules, and corneal inflammation with azithromycin treatment.** (**A**) Evolution of bulbar conjunctival hyperemia (number of eyes). (**B**) Evolution of conjunctival phlyctenules (number of eyes). (**C**) Evolution of corneal inflammation (number of eyes).

The treatment and follow-up durations per patient are reported in Table 2. Topical azithromycin was administered for a mean of 6.0 ± 1.4 months (range, 4 to 10 months). Patients received three treatments of azithromycin per month for a mean duration of 2 months (range, 1 to 6 months), then two treatments per month for a mean of 2 months (range, 1 to 2 months), then one treatment per month for a mean of 2 months (range, 0 to 2 months). The disease was controlled by topical azithromycin alone in 15 out of 16 patients (18 eyes). The remaining patient (one eye) required the addition of cyclosporine 2% eye drops for 5 months after 5 months of azithromycin in order to control ocular inflammation. Topical azithromycin dramatically improved ocular redness (reported by the patient) and bulbar conjunctival hyperemia (assessed by the ophthalmologist), both of which resolved within 1 month in all eyes (Figure 1A). Conjunctival phlyctenules and corneal inflammation took longer to improve, with a complete resolution within 3 to 10 months (Figure 1B,C). Inferior superficial punctate keratitis only partially resolved in all eyes.

**Table 2:** Treatment, follow-up duration, and adverse events

**Patient no.**	**Treatments associated with azithromycin**	**Azithromycin treatment duration (months)**	**Follow-up without azithromycin (months)**	**Adverse event**
1	LH	6	8	
2	LH	4	9	
3	LH	6	12	Moderate ocular redness upon instillation
4	LH, topical cyclosporine added at month 5 for 5 months	10	11	Important burning, treatments stopped at month 10
5	LH	6	9	Mild stinging upon instillation
6	LH	5	13	
7	LH	6	9	
8	LH	8	12	
9	LH	4	10	Mild stinging upon instillation
10	LH	6	11	
11	LH	6	9	
12	LH	6	12	Moderate ocular redness upon instillation
13	LH	6	10	
14	LH	6	11	
15	LH	6	12	
16	LH	5	14	Important burning, treatment stopped at month 5

LH, lid hygiene.

Blepharitis grading was available in 13 patients (16 eyes). Mean grade decreased from 2.31 ± 0.79 at baseline to 1.50 ± 0.73 at the end of the treatment. Although anterior and posterior signs of blepharitis were not graded separately, anterior blepharitis seemed to improve more significantly than posterior blepharitis and meibomian gland dysfunction. However, no episodes of chalazion were noted during azithromycin treatment, even in the two children who complained of recurrent chalazia at baseline.

Azithromycin was stopped when conjunctival phlyctenules and corneal inflammation had completely resolved, which occurred after a mean of 6.0 ± 1.4 months (between 4 and 10 months). Corneoconjunctival inflammation did not recur with lid hygiene alone after a mean follow-up of 11.0 ± 1.7 months (8 to 14 months).

### Adverse events

Adverse events are summarized in Table 2. Mild sensations of ocular stinging upon instillation were reported in two patients and moderate ocular redness after instillation in two other cases. Treatment was stopped because of local intolerance (significant sensation of burning) in two other patients after 5 and 10 months. As the disease was already controlled for these last two patients, no recurrences were observed after azithromycin was withdrawn. No systemic adverse events were reported by the parents.

## Discussion

Childhood ocular rosacea is believed to be the consequence of chronic meibomian gland dysfunction. Secondary staphylococcal infection of the lid margin is often present and is probably responsible for the corneoconjunctival inflammation by means of an immunological specific T cell response. This combined mechanism explains the efficacy of both topical anti-inflammatory drugs (steroids or cyclosporine) and systemic antibiotics such as macrolides or tetracyclines.

Azithromycin is a new treatment option for cutaneous rosacea and seems to be as effective as tetracyclines [13], and its efficacy in treating ocular rosacea has been demonstrated when administered orally [12].

Azithromycin eye drops are now available for the treatment of bacterial conjunctivitis, and in this indication, azithromycin 1.5% eye drops given twice daily for 3 days is as effective as tobramycin 0.3% eye drops four times a day for 7 days [14].

Our study showed that topical azithromycin 1.5% is a very effective treatment of ocular rosacea in children. Its efficacy on conjunctival and corneal inflammation is remarkable. However, because of the delayed action on corneal inflammation, prior clinical experience has shown that very severe cases with vision threat should be concomitantly treated with topical steroids and/or cyclosporine. Oral erythromycin is usually prescribed in childhood ocular rosacea [15]. In this study, in one patient who had been unsuccessfully treated with oral erythromycin, replacement by azithromycin resulted in a complete control of inflammation. Our previous treatment strategy was to use oral erythromycin as a first-line treatment in combination with lid hygiene. However, our early experience with topical azithromycin showed us that this local treatment was superior to systemic erythromycin. This is why we decided to use, since then, topical azithromycin as a first-line therapy. Topical azithromycin has several advantages over oral antibiotics. Azithromycin has a very long half-life, with significant tissular accumulation and concentrations. In a rabbit model, after administration of 1% azithromycin ophthalmic solution, significant concentrations are detected in the tears, conjunctiva, cornea, and lids for as long as 6 days in the lids [10]. Concentrations in the tears, conjunctiva, and cornea remained above minimal inhibitory concentrations for respectively 7, 17, and more than 24 days after instillation of Azyter® (Thea Laboratories, Clermont-Ferrand, France) twice a day (bid) for 3 days [16]. This is why we chose a discontinuous treatment regimen which was very simple for the patients; one treatment corresponds to one box of eye drops, and so the initial scheme of three treatments per month tapered to two and then one per month was convenient, easy to understand, and well accepted by the children and their parents. The initial dosing (3 days three times monthly) was chosen because of the 7-day persistence of azithromycin in the tears. The tapering scheme was chosen empirically. Oral erythromycin has to be taken twice daily for a continuous period of several weeks. In the 16 patients studied, topical azithromycin did not induce systemic side effects, whereas oral erythromycin frequently induces gastrointestinal troubles. Oral metronidazole has also been proposed as a treatment for ocular rosacea, but while it seems to be an effective treatment, the fact that short-course therapy is required in order to avoid peripheral neuropathy may result in more frequent relapses [6].

Few publications reported the efficacy of topical antibiotics in this disease. In a retrospective study by Viswalingam et al., topical chloramphenicol eyedrops four times daily for 1 month and chloramphenicol ointment at night for 4 months were successfully prescribed in mild to moderate cases [3]. Azithromycin ophthalmic solution 1% has been evaluated in posterior blepharitis in adults in two small studies [17,18]. In one study, meibum quality and lid margin redness improved more in patients treated with lid hygiene and azithromycin for 2 weeks than in controls treated with only lid hygiene. In the second study, symptoms, meibum quality, and lid margin redness improved after treatment with lid hygiene and azithromycin for 4 weeks. While we did not specifically analyze the posterior lid margin in our study, the effect of topical azithromycin on anterior blepharitis was noticeable, and no chalazia were noted.

Topical steroids are frequently prescribed in children with ocular rosacea and phlyctenular blepharokeratoconjunctivitis, but relapses occur after the end of the treatment in up to 40% of cases [15]. Treatment duration is a key element for avoiding recurrences. In our study, treatment was stopped after 4 to 10 months, and no recurrence was observed during a median follow-up of 11 months (8 to 14 months). Long-term treatments are usually required to control the inflammation. A complete disappearance of the phlyctenules and corneal infiltrates is necessary before stopping the treatment, which was attained in 3 to 6 months in the majority of children included in this study with topical azithromycin. This minimal treatment duration is comparable with topical cyclosporine [9] or topical chloramphenicol [3]. Such duration is not acceptable with topical steroids because of the risk of ocular complications. In our series, topical steroids were withdrawn in all patients when topical azithromycin was prescribed, and none of the patients required additional steroid therapy thereafter.

In a previous study, we showed that topical 2% cyclosporine is a very potent treatment of phlyctenular blepharokeratoconjunctivitis [9]. The efficacy of topical azithromycin was sufficient in most cases, but as clinical experience indicates that topical cyclosporine may have greater efficacy, patients with very severe corneal inflammation were prescribed topical cyclosporine as first-line treatment.

A biphasic effect was observed in this study. The effect on ocular redness was very fast, within a month, whereas phlyctenules and corneal infiltrates took several months to heal. This may point to different mechanisms. Ocular redness might be related to bacterial toxin production. Phlyctenules and corneal infiltrates are thought to be caused by a type IV cell-mediated delayed hypersensitivity reaction against parietal staphylococcal antigens [5]. In addition to its antibiotic effect, azithromycin also has anti-inflammatory effects. Indeed, it has been shown to reduce the production of the pro-inflammatory cytokines IL-12 and IL-6 by macrophages *in vitro*[11]; to suppress matrix metalloproteases 2 and 9, nuclear transcription factor NFkB, and Toll-like receptor 2 in corneal epithelial cells *in vitro*[19,20]; and to inhibit macrophage and dendritic cell migration after corneal burn in a mouse model [21].

The safety profile of topical 1.5% azithromycin seems good in children suffering from ocular rosacea. Discontinuous treatment with topical 1.5% azithromycin was well tolerated. Local intolerance was seen in a small number of cases and was usually mild, except in two patients who stopped the treatment after 5 and 10 months, respectively. Comparable side effects have been reported in the literature [17,18].

The main limitation of our study is the small number of patients, but rosacea in children is infrequent, and the largest series published to date included 44 children [3]. The retrospective nature of this study is also an important limitation. A multicenter prospective controlled study should be the next step for evaluating azithromycin eyedrops in childhood ocular rosacea.

## Conclusion

In conclusion, this retrospective study showed that topical 1.5% azithromycin eye drops is an effective and safe treatment in non-severe forms of childhood ocular rosacea with phlyctenular blepharokeratoconjunctivitis. When treated under a discontinuous but prolonged regimen for at least 4 months, recurrences did not occur up to 14 months after the end of treatment. Considering the benefits/risk ratio, this new treatment could probably replace oral antibiotics in this disease, but further controlled studies are required.

## Methods

### Design

This is a retrospective, non-comparative, interventional case series.

### Methods

We reviewed the files of all 16 consecutive children with rosacea and phlyctenular blepharokeratoconjunctivitis who were treated with topical 1.5% azithromycin eye drops between January 2009 and March 2010 in the Department of Ophthalmology at Bichat Hospital and Fondation A. de Rothschild, Paris, France. Written informed consent was obtained from the patients and from their parents for publication of this report. This study was approved by the Ethics Committee of Foundation A de Rothschild.

### Treatment strategy

Patients presenting with ocular rosacea were initially treated with lid hygiene once daily, and frequent normal saline instillations were initially prescribed alone for at least 1 month. Patients with sight-threatening corneal infiltrates approaching the visual axis were treated with steroids and cyclosporine 2% eye drops and were not treated with azithromycin; therefore, they were not included in the study.

Azithromycin 1.5% eye drops (Azyter®, Thea Laboratories) was administered only to patients who did not respond to lid hygiene. A three-day treatment (1 drop bid) was initially prescribed every 10 days (i.e., three treatments monthly) for at least 1 month. According to clinical efficacy, treatment was tapered to one treatment every 15 days and then one treatment monthly.

### Follow-up

Patients were examined after 1 month and every 1 to 2 months thereafter.

The main outcome measures were ocular redness (reported by the patients), occurrence of chalazia (reported by the patients), conjunctival bulbar hyperemia, conjunctival phlyctenules, corneal inflammation (phlyctenules and infiltrates) and epitheliopathy, and lid margin inflammation (blepharitis). All measures were graded on scales of 0 to 4. Ocular rosacea was considered to be controlled if all items except lid margin inflammation had a score of 0. Ocular and systemic adverse events were also reported.

## Competing interest

SD, FC, and IC are consultants for Thea Laboratories. However, they did not receive any financial support for this study.

## Authors’ contributions

SD designed the study, gathered the data, performed the data analysis, and wrote the manuscript. EG, MT, FC, and IC contributed to the data analysis and discussion. All authors read and approved the final manuscript.

## References

[B1] Thygeson P The etiology and treatment of phlyctenular keratoconjunctivitis Am J Ophthalmol 1951 34 9 1217 1236 14877944 10.1016/0002-9394(51)91857-0

[B2] Farpour B McClellan KA Diagnosis and management of chronic blepharokeratoconjunctivitis in children J Pediatr Ophthalmol Strabismus 2001 38 4 207 212 11495307 10.3928/0191-3913-20010701-06

[B3] Viswalingam M Rauz S Morlet N Dart JK Blepharokeratoconjunctivitis in children: diagnosis and treatment Br J Ophthalmol 2005 89 4 400 403 10.1136/bjo.2004.052134 15774912 PMC1772603

[B4] Doan S Gabison EE Nghiem-Buffet S Abitbol O Gatinel D Hoang-Xuan T Long-term visual outcome of childhood blepharokeratoconjunctivitis Am J Ophthalmol 2007 143 3 528 529 10.1016/j.ajo.2006.09.058 17317407

[B5] Mondino BJ Kowalski RP Phlyctenulae and catarrhal infiltrates. Occurrence in rabbits immunized with staphylococcal cell walls Arch Ophthalmol 1982 100 12 1968 1971 10.1001/archopht.1982.01030040948017 7150063

[B6] Chamaillard M Mortemousque B Boralevi F da CC M Aitali F Taieb A Léauté-Labrèze C Cutaneous and ocular signs of childhood rosacea Arch Dermatol 2008 144 2 167 171 10.1001/archdermatol.2007.50 18283173

[B7] Zaidman GW Brown SI Orally administered tetracycline for phlyctenular keratoconjunctivitis Am J Ophthalmol 1981 92 2 178 182 7270629 10.1016/0002-9394(81)90766-2

[B8] Meisler DM Raizman MB Traboulsi EI Oral erythromycin treatment for childhood blepharokeratitis J AAPOS 2000 4 6 379 380 10.1067/mpa.2000.110339 11124676

[B9] Doan S Gabison E Gatinel D Duong MH Abitbol O Hoang-Xuan T Topical cyclosporine a in severe steroid-dependent childhood phlyctenular keratoconjunctivitis Am J Ophthalmol 2006 141 1 62 66 10.1016/j.ajo.2005.08.035 16386977

[B10] Akpek EK Vittitow J Verhoeven RS Brubaker K Amar T Powell KD Boyer JL Crean C Ocular surface distribution and pharmacokinetics of a novel ophthalmic 1% azithromycin formulation J Ocul Pharmacol Ther 2009 25 5 433 439 10.1089/jop.2009.0026 19857105

[B11] Murphy BS Sundareshan V Cory TJ Hayes D Jr Anstead MI Feola DJ Azithromycin alters macrophage phenotype J Antimicrob Chemother 2008 61 3 554 560 10.1093/jac/dkn007 18230686

[B12] Bakar O Demircay Z Toker E Cakir S Ocular signs, symptoms and tear function tests of papulopustular rosacea patients receiving azithromycin J Eur Acad Dermatol Venereol 2009 23 5 544 549 10.1111/j.1468-3083.2009.03132.x 19250326

[B13] Akhyani M Ehsani AH Ghiasi M Jafari AK Comparison of efficacy of azithromycin vs. doxycycline in the treatment of rosacea: a randomized open clinical trial Int J Dermatol 2008 47 3 284 288 10.1111/j.1365-4632.2008.03445.x 18289334

[B14] Cochereau I Meddeb-Ouertani A Khairallah M Amraoui A Zaghloul K Pop M Delval L Pouliquen P Tandon R Garg P Goldschmidt P Bourcier T 3-day treatment with azithromycin 1.5% eye drops versus 7-day treatment with tobramycin 0.3% for purulent bacterial conjunctivitis: multicentre, randomised and controlled trial in adults and children Br J Ophthalmol 2007 91 4 465 469 10.1136/bjo.2006.103556 17050578 PMC1994738

[B15] Hammersmith KM Cohen EJ Blake TD Laibson PR Rapuano CJ Blepharokeratoconjunctivitis in children Arch Ophthalmol 2005 123 12 1667 1670 10.1001/archopht.123.12.1667 16344437

[B16] Amar T Caillaud T Elena PP Ocular pharmacokinetic study following a single and multiple azithromycin administrations in pigmented rabbits Curr Eye Res 2008 33 2 149 158 10.1080/02713680701860499 18293185

[B17] Luchs J Efficacy of topical azithromycin ophthalmic solution 1% in the treatment of posterior blepharitis Adv Ther 2008 25 9 858 870 10.1007/s12325-008-0096-9 18781287

[B18] Haque RM Torkildsen GL Brubaker K Zink RC Kowalski RP Mah FS Pflugfelder SC Multicenter open-label study evaluating the efficacy of azithromycin ophthalmic solution 1% on the signs and symptoms of subjects with blepharitis Cornea 2010 29 8 871 877 10.1097/ICO.0b013e3181ca38a0 20508503

[B19] Jacot JL Jacot TA Hahto S Helis J Sheppard CJ Sheppard JD Jr Lattanzio FA Jr Williams PB Azithromycin alters ProMMP-2 and TIMP-1 following corneal wounding in an experimental animal model of diabetic ocular complications Invest Ophthalmol Vis Sci 2009 50 5 E-Abstract 2663

[B20] Li D-Q Zhou N Zhang L Ma P Pflugfelder SC Suppressive effects of azithromycin on zymosan-induced production of proinflammatory mediators by human corneal epithelial cells Invest Ophthalmol Vis Sci 2010 51 11 5623 5629 10.1167/iovs.09-4992 20538995 PMC3061501

[B21] Sadrai Z Hajrasouliha AR Chauhan S Saban D Dana R Anti-inflammatory activity of topical azithromycin ophthalmic solution 1% in the treatment of ocular inflammation Invest Ophthalmol Vis Sci 2010 51 E-Abstract 3789

